# Final Analysis of the Phase 1/2 Trial of Valoctocogene Roxaparvovec for Severe Haemophilia A

**DOI:** 10.1111/hae.70284

**Published:** 2026-04-12

**Authors:** Priyanka Raheja, Savita Rangarajan, Will Lester, Bella Madan, Glenn F. Pierce, Emily Symington, Carolyn Millar, Dane Osmond, Mingjin Li, Konstantia‐Maria Chavele

**Affiliations:** ^1^ Haemophilia Centre Barts Health NHS Trust, The Royal London Hospital London UK; ^2^ Faculty of Medicine University of Southampton Southampton UK; ^3^ University Hospitals Birmingham NHS Foundation Trust Birmingham UK; ^4^ Guy's and St Thomas’ NHS Foundation Trust London UK; ^5^ Independent Consultant, La Jolla San Diego California USA; ^6^ Haemophilia Centre Cambridge University Hospitals NHS Foundation Trust Cambridge UK; ^7^ Imperial College Healthcare NHS Trust and Centre for Haematology, Department of Immunology and Inflammation Imperial College London London UK; ^8^ BioMarin Pharmaceutical Inc. Novato California USA

**Keywords:** adeno‐associated virus, clinical trial, factor VIII, gene therapy, Haemophilia

## Abstract

**Introduction:**

Valoctocogene roxaparvovec is an adeno‐associated virus vector serotype 5 (AAV5)‐mediated gene therapy for severe haemophilia A (HA).

**Aim:**

Report the final safety and efficacy results of the phase 1/2 trial of valoctocogene roxaparvovec.

**Methods:**

An open‐label phase 1/2 trial (NCT02576795) enrolled adult males with severe HA (factor VIII [FVIII] ≤1 IU/dL) without FVIII inhibitors, anti‐AAV5 antibodies, or liver dysfunction to receive 6 × 10^13^ or 4 × 10^13^ vg/kg valoctocogene roxaparvovec. Efficacy endpoints included FVIII activity (chromogenic), annualized bleeding rate (ABR) of treated bleeds, annualized FVIII infusion rate, and quality of life. Safety was assessed by adverse events (AEs). Year seven 6 × 10^13^ vg/kg cohort results were reported previously.

**Results:**

Five of six 4 × 10^13^ vg/kg cohort participants completed the 7‐year study. Mean FVIII activity peaked within year 1 for the 4 × 10^13^ cohort (week 52 FVIII, 21.1 IU/dL) and decreased to 4.2 IU/dL at the end of year 7. Across all follow‐up, mean ABR declined 87% from baseline to 1.6 bleeds/year and mean annualized FVIII use declined 93% to 10.3 infusions/year. Overall, 3/5 of the 4 × 10^13^ participants remain off prophylaxis. Haemo‐QOL‐A Total Score was mostly stable from baseline. The last treatment‐related AE (TRAE) of alanine aminotransferase elevation occurred in year 1. No serious TRAEs occurred after year 1. No participants had thromboembolic events or developed FVIII inhibitors.

**Conclusion:**

Over 7 years, valoctocogene roxaparvovec increased FVIII activity from baseline and improved haemostasis compared with FVIII prophylaxis for most participants. The 6 × 10^13^ vg/kg dose was more efficacious than 4 × 10^13^ vg/kg. No concerning long‐term safety signals were identified.

## 1 Introduction

Haemophilia A (HA) is caused by low levels of the blood‐clotting protein factor VIII (FVIII) [1]. Treatment guidelines for severe HA, classified as FVIII activity <1 IU/dL, recommend regular prophylaxis with exogenous FVIII or emicizumab [1]. The treatment burden related to the chronic infusions or subcutaneous injections required for these forms of prophylaxis can diminish health‐related quality of life (HRQOL) [1, 2].

Valoctocogene roxaparvovec is a gene therapy approved in the United States and European Union for the treatment of severe HA that enables endogenous FVIII production using a liver‐directed adeno‐associated virus serotype 5 (AAV5) vector to transfer a B‐domain‐deleted human FVIII coding sequence controlled by a hepatocyte‐selective promoter [3, 4, 5, 6, 7, 8, 9, 10]. To investigate the safety, tolerability, and efficacy of valoctocogene roxaparvovec, a phase 1/2 trial with a follow‐up period of 7 years was planned to sequentially enrol participants to receive a single intravenous dose of 6 × 10^12^, 2 × 10^13^, 6 × 10^13^, and 4 × 10^13^ vg/kg valoctocogene roxaparvovec (NCT02576795). This and a later phase 3 (NCT03370913; GENEr8‐1) trial have both demonstrated the efficacy and safety of valoctocogene roxaparvovec in participants with severe HA [5, 6, 9, 10, 11, 12, 13, 14, 15]. In both trials, most participants had sustained FVIII activity and reduced bleeding rates, no participants developed FVIII inhibitors, and the most common adverse events (AEs) were asymptomatic alanine aminotransferase (ALT) elevations, which were effectively managed using glucocorticoids [5, 6, 9, 10, 11, 12, 13, 14, 15].

Safety and efficacy data for the 6 × 10^13^ vg/kg cohort of the phase 1/2 trial were previously reported for the full 7 years of follow‐up [14]. Briefly, one participant in the 6 × 10^13^ cohort reported a treatment‐related AE of grade 1 hepatomegaly in year 7 [14]. At year 7, mean (median) FVIII activity (chromogenic substrate assay [CSA]) was 16.2 (10.3) IU/dL with an estimated rate of change in FVIII activity of −0.001 IU/dL/week in the last year [14]. In year 7, 1 participant resumed prophylaxis after a non‐treatment‐related grade 4 serious AE of spontaneous internal carotid artery bleed and another resumed prophylaxis to manage bleeds [14]. The safety and efficacy of valoctocogene roxaparvovec remained generally consistent with previous reports [14].

Here, we present the final results from the phase 1/2 trial, including safety and efficacy data from participants in the 4 × 10^13^ vg/kg cohort who completed the seventh and final year of the trial. We also report HRQOL outcomes for both the 4 × 10^13^ vg/kg and 6 × 10^13^ vg/kg cohorts. Finally, we provide a retrospective discussion of the entirety of the trial, including comparisons with the phase 3 GENEr8‐1 study and insights into the long‐term benefit of valoctocogene roxaparvovec gene therapy for severe HA.

## 2 Methods

### 2.1 Study Design

The design of this open‐label, phase 1/2 dose‐escalation trial (NCT02576795) has been described previously [6, 9, 11, 16]. Briefly, males ≥18 years of age with severe HA (FVIII ≤1 IU/dL) who were previously receiving exogenous FVIII received an infusion of 6 × 10^12^ (*n* = 1), 2 × 10^13^ (*n* = 1), 4 × 10^13^ (*n* = 6), or 6 × 10^13^ (*n* = 7) vg/kg valoctocogene roxaparvovec [6]. Efficacy and safety data from participants in the 4 × 10^13^ vg/kg cohort across 7 years of follow‐up are reported here; results in the 6 × 10^13^ vg/kg cohort after 7 years were published previously [14]. Final results from participants who received 6 × 10^12^ and 2 × 10^13^ vg/kg were also published previously [6]. Additionally, HRQOL outcomes are reported for the 4 × 10^13^ vg/kg and 6 × 10^13^ vg/kg cohorts. Participants did not have a history of FVIII inhibitors, anti‐AAV5 antibodies, significant liver dysfunction, significant liver fibrosis, or liver cirrhosis [6, 9, 11, 16].

### 2.2 Assessments

Safety was assessed with physical examinations, laboratory assessments, and by the documentation of AEs graded with Common Terminology Criteria for Adverse Events v4.03. Annualized treated bleeding rates (ABRs) and annualized FVIII infusion rates were calculated as described previously [6, 9]. Baseline ABRs were derived from the 12 months prior to enrolment. FVIII activity was assessed via CSA and one‐stage assay (OSA) and are reported excluding data from participants who resumed prophylaxis. FVIII measurements were not considered valid within 72 h of exogenous FVIII use. Participants completed the Haemophilia‐Specific Quality of Life Questionnaire for Adults (Haemo‐QOL‐A) at baseline and periodically throughout the study; higher scores indicate better HRQOL [17]. Haemo‐QOL‐A results are reported for both the 4 × 10^13^ vg/kg and 6 × 10^13^ vg/kg cohorts.

### 2.3 Statistics

Data are presented with descriptive statistics. Missing data were not imputed. The yearly rate of change in FVIII activity was calculated using a linear regression model as FVIII = intercept + slope*week (random intercept and slope). Data following resumption of prophylaxis were excluded for FVIII activity endpoints and included for ABR, annualized FVIII infusion rate, and Haemo‐QOL‐A endpoints.

## 3 Results

### 3.1 Participants

All 6 participants in the 4 × 10^13^ vg/kg cohort were receiving FVIII prophylaxis before enrolment. Demographics and baseline disease characteristics were published previously [9]. As of the database lock, 5 of 6 (83.3%) participants in the 4 × 10^13^ vg/kg cohort completed the study and 1 (16.7%) participant was lost to follow‐up after week 288.

### 3.2 Safety

In year 7, 2 participants in the 4 × 10^13^ vg/kg cohort experienced 1 AE each (Table 1). Neither AE was determined to be treatment‐related or classified as serious. One AE was an ALT elevation at week 336 that was not resolved by the database lock; the participant who experienced this event had a history of fatty liver disease. The other AE was viral infection. No new safety signals were identified.

**TABLE 1:** Summary of incidence of AEs in the 4 × 10^13^ vg/kg cohort.

	Y1	Y2	Y3	Y4	Y5	Y6	Y7
Any AE	6 (100)	5 (83.3)	5 (83.3)	4 (66.7)	6 (100)	4 (66.7)	2 (33.3)
Any SAE	1 (16.7)	0	1 (16.7)	1 (16.7)	1 (16.7)	0	0
Any treatment‐related AE	6 (100)	0	0	0	1 (16.7)	1 (16.7)	0
Any treatment‐related SAE	1 (16.7)^a^	0	0	0	0	0	0
AEs of special interest							
ALT elevation^b^	4 (66.7)	0	1 (16.7)	0	0	0	0^c^
AEs of liver dysfunction^d^	5 (83.3)	0	1 (16.7)	0	0	0	1 (16.7)
Potential Hy's law case	0	0	0	0	0	0	0
Infusion‐related reactions	4 (66.7)	0	0	0	0	0	0
Systemic hypersensitivity	0	0	0	0	0	0	0
Anaphylactic or anaphylactoid reactions	0	0	0	0	0	0	0
Thromboembolic events	0	0	0	0	0	0	0

*Note*: Data are presented as *n* (%).

Abbreviation: AE, adverse event; ALT, alanine aminotransferase; CTCAE, Common Terminology Criteria for Adverse Events; MedDRA, Medical Dictionary for Regulatory Activities; SAE, serious AE; ULN, upper limit of normal; Y, year.

^a^ Pyrexia on study day 2.

^b^ Defined as ALT ≥1.5x ULN or ALT ≥1.5x baseline.

^c^ Participant 10 experienced a non‐related CTCAE grade 1 ALT elevation in year 7 that was ongoing at the time of the database lock; this event was not coded as an AE of special interest and was thus not included in the main tabulation.

^d^ Identified with a MedDRA search strategy using the high‐level term “liver function analyses.”

### 3.3 Efficacy

**FIGURE 1:**
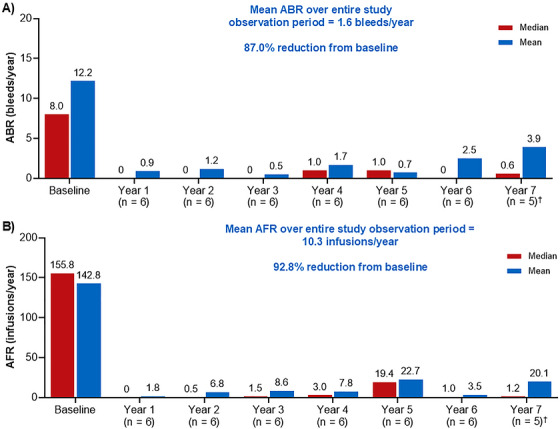
(A) Annualized bleeding rates for treated bleeds and (**B)** annualized FVIII infusion rates in the 4 × 10^13^ vg/kg cohort. Data included participants who resumed prophylaxis. ^†^One participant lost to follow‐up. ABR, annualized bleeding rate; AFR, annualized FVIII infusion rate; FVIII, factor VIII.

In year 7, the mean and median ABR for treated bleeds were 3.9 bleeds/year and 0.6 bleeds/year, respectively, in the 4 × 10^13^ vg/kg cohort (Figure 1A). Across the entire study, the mean ABR for treated bleeds was 1.6 bleeds/year (median, 0.5 bleeds/year), an 87.0% decrease from baseline. Mean and median annualized FVIII infusion rates in year 7 were 20.1 infusions/year and 1.2 infusions/year, respectively (Figure 1B). Individual bleeding and FVIII data for all participants in the 4 × 10^13^ vg/kg and 6 × 10^13^ vg/kg cohorts are provided in Table S1. Over the entire 7‐year follow‐up, 3 of the 7 participants in the 6 × 10^13^ vg/kg cohort experienced 0 treated bleeds, and 2 of 7 participants in the 6 × 10^13^ vg/kg cohort and 2 of 6 participants in the 4 × 10^13^ vg/kg cohort resumed prophylaxis.

**FIGURE 2:**
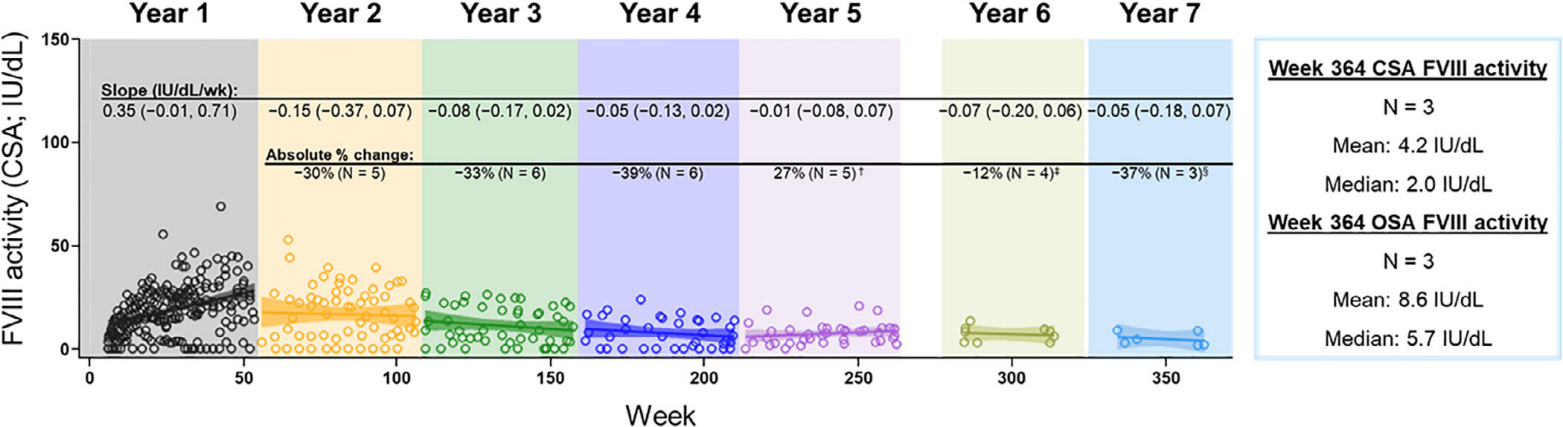
FVIII activity over 7 years in the 4 × 10^13^ vg/kg cohort. Data excluded participants who resumed prophylaxis. Slope and error are mean (95% CI). ^†^One participant resumed prophylaxis. ^‡^Two participants resumed prophylaxis. ^§^Two participants resumed prophylaxis; one participant lost to follow‐up. CI, confidence interval; CSA, chromogenic substrate assay; FVIII, factor VIII; OSA, one‐stage assay.

Mean FVIII activity per CSA was 4.2 IU/dL (median, 2.0 IU/dL) at week 364 in the 4 × 10^13^ vg/kg cohort, with a slope of −0.05 IU/dL/week (95% confidence interval, −0.18 to 0.07) estimated during year 7 (Figure 2). The absolute percent decrease in mean FVIII activity per CSA was 37.3% between year 6 and year 7. Of the 3 participants who had not resumed prophylaxis and continued in the study, 1 had FVIII activity per CSA in the mild haemophilia range (>5 to 40 IU/dL) and 2 had FVIII activity in the moderate haemophilia range (1.5 to 5 IU/dL). Participant 15, who did not return to prophylaxis per the protocol definition but did use regular prophylaxis transiently over 3 weeks, had FVIII activity below the lower limit of quantification (<1.5 IU/dL) at the end of year 7.

In year 7, 3 participants in the 4 × 10^13^ vg/kg cohort experienced 20 treated bleeds. Participant 10 had a traumatic treated thigh bleed that started in week 357 that was caused by kicking a football and lasted 17 days. The most recent FVIII activity for this participant, assessed 19 weeks before the event, was 3.0 IU/dL per CSA and 6.6 IU/dL per OSA. Participant 11, who had 15 ankle bleeds in the sixth year of follow‐up due to chronic bilateral ankle arthropathy as previously reported, had 14 ankle bleeds in year 7 and resumed prophylaxis with exogenous FVIII during week 335 [14]. Ten of the 14 ankle bleeds occurred after resuming prophylaxis, 6 of which were spontaneous and 4 of which were traumatic. The most recent FVIII activity for this participant (9.4 IU/dL per CSA; 17.0 IU/dL per OSA) was assessed 22 weeks prior to resuming prophylaxis. Participant 15 had treated bleeds in the leg (1), knee (1), ankle (2), and shoulder (1). Three of these bleeds were caused by trauma due to a fall, leaning on his arm, or periods of heightened activity. One of the remaining 2 bleeds was spontaneous; it is unknown whether the other was spontaneous or traumatic. This participant had previously resumed prophylaxis transiently during year 5 and used FVIII concentrate on multiple occasions during year 7, including for short‐term prophylaxis before left elbow surgery, transient routine prophylaxis, precautionary prophylaxis due to activity and injury, and for the treatment of shoulder, knee, leg, and ankle bleeds. No valid FVIII activity results were available for this participant during year 7 due to his use of exogenous FVIII (median FVIII activity in year 5, ≤1.5 IU/dL per CSA and 2.4 to 3.1 IU/dL per OSA).

### 3.4 Haemo‐QOL‐A

**FIGURE 3:**
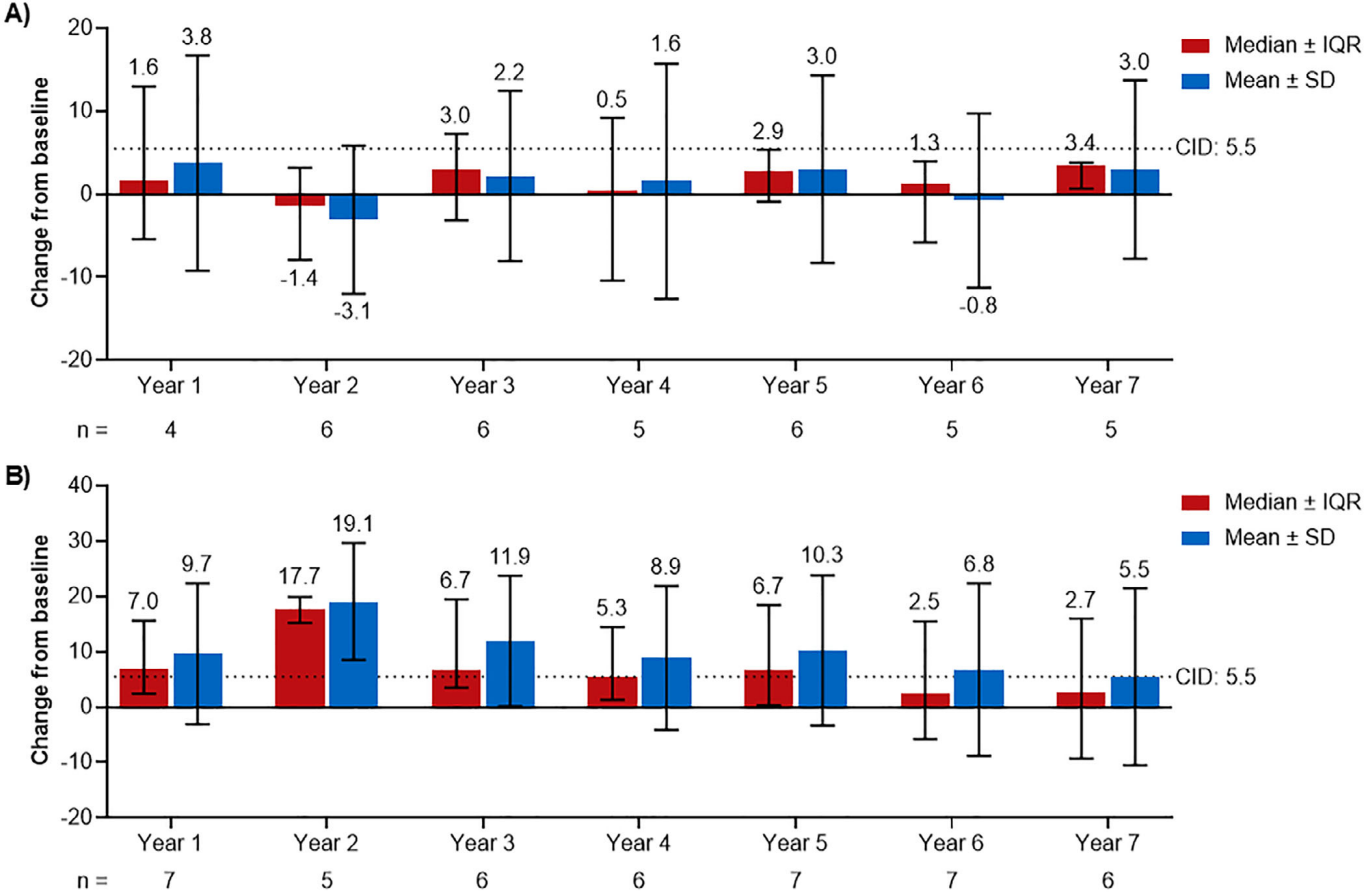
Change from baseline in Haemo‐QOL‐A Total Score in the (**A)** 4 × 10^13^ vg/kg cohort and (**B)** 6 × 10^13^ vg/kg cohort. Data included participants who resumed prophylaxis. CID, clinically important difference; Haemo‐QOL‐A, Haemophilia‐Specific Quality of Life Questionnaire for Adults; IQR, interquartile range; SD, standard deviation.

In the 4 × 10^13^ vg/kg cohort (*n* = 6), mean changes from baseline in Haemo‐QOL‐A Total Score at the end of years 5 (3.0), 6 (−0.8), and 7 (3.0) were generally stable from the mean (standard deviation [SD]) baseline score of 80.8 (9.1) (Figure 3A) [5]. Data from participants who resumed prophylaxis are included in the Haemo‐QOL‐A results. In this cohort, the domain score of Treatment Concern was most improved at the end of year 7 compared with baseline (8.0), though no clinically important difference (CID) has been established for this domain [17]. The positive changes from baseline in the other domain scores of Worry (5.6), Role Functioning (1.8), and Physical Functioning (1.3; Figure S1) did not meet the anchor‐based CID of 6.0 [17]. The domain scores of Consequences of Bleeding and Emotional Impact were unchanged from baseline.

In the 6 × 10^13^ vg/kg cohort (*n* = 7), mean Haemo‐QOL‐A Total Score increased 6.8 points from baseline (mean [SD], 71.9 [16.6]) at the end of year 6 (Figure 3B). From baseline to the end of year 7, the mean Total Score increased 5.5 points, which met the CID of 5.5 for Total Score [17]. In this cohort, the greatest mean increases from baseline to the end of year 7 were in the domain scores of Role Functioning (10.3), Emotional Impact (10.0), and Consequences of Bleeding (6.7), which all met the CID of 6.0, followed by Worry (5.3) and Physical Functioning (4.1; Figure S2) [17]. The Treatment Concern domain score decreased from baseline to the end of year 7 (−3.3).

## 4 Discussion

In the seventh and final year of follow‐up for this study, results were mostly the same as the previous year, with no new serious or treatment‐related safety events and continued improved protection from bleeding compared with baseline for most participants. With the completion of follow‐up for both the 4 × 10^13^ vg/kg and 6 × 10^13^ vg/kg cohorts, we present a retrospective narrative of results and insights from across the trial.

Over 7 years following a single infusion, valoctocogene roxaparvovec provided clinically relevant improvements in haemostatic efficacy compared with baseline and an acceptable safety profile [5, 6, 9, 11, 14]. The most common AEs were ALT elevations, the incidence of which peaked in year 1 for both the 4 × 10^13^ vg/kg and 6 × 10^13^ vg/kg cohorts [14]. As in GENEr8‐1, most ALT elevations were asymptomatic, low‐grade, and manageable with corticosteroids [10, 12, 13, 15]. One treatment‐related AE of worsening hepatic steatosis occurred in a 4 × 10^13^ vg/kg cohort participant with a history of fatty liver disease. Other liver‐related AEs included instances of hepatomegaly (6 × 10^13^ vg/kg cohort) and splenomegaly (4 × 10^13^ vg/kg cohort); in both cases, relatedness to treatment could not be ruled out [14]. No thromboembolic events or anaphylactoid reactions occurred in the study [5, 6, 9, 11, 14]. No events of systemic hypersensitivity occurred, in contrast to the phase 3 GENEr8‐1 study, in which 5.2% of participants experienced hypersensitivity [6, 9, 10]. In the 4 × 10^13^ vg/kg cohort, the lower dose compared with the 6 × 10^13^ vg/kg administered in GENEr8‐1 may have reduced the risk of hypersensitivity, whereas the small size of the 6 × 10^13^ vg/kg cohort in the phase 1/2 trial may have prevented detection of this relatively infrequent AE.

One case of acinar cell carcinoma occurred in a 6 × 10^13^ vg/kg cohort participant 6 years after treatment, but extensive genomic analyses determined that the event was not related to valoctocogene roxaparvovec [11]. In response to this event, this study employed the first detailed methodology and analysis strategy for determining the relatedness of tumorigenesis to an AAV gene therapy for HA [11]. A tumorigenic event also occurred in GENEr8‐1 (a B‐cell acute lymphoblastic leukaemia diagnosis in year 3), which was likewise determined to be unrelated to treatment [15]. No cancers have been attributed to AAV gene therapies for any indication to date in humans [18, 19, 20].

FVIII activity for both cohorts peaked by the end of year 1 and was higher in the 6 × 10^13^ cohort than the 4 × 10^13^ cohort, with mean week 52 FVIII of 63.6 IU/dL (median, 60.3 IU/dL) and 21.1 IU/dL (median, 23.8 IU/dL), respectively. After decreasing, final mean FVIII levels were 16.2 IU/dL (median, 10.3 IU/dL) in the 6 × 10^13^ cohort and 4.2 IU/dL (median, 2.0 IU/dL) in the 4 × 10^13^ cohort at the end of 7 years [14]. This remaining endogenous FVIII production was associated with fewer than 1 bleed/year on average in the 6 × 10^13^ vg/kg cohort. Importantly, while the general trajectory of FVIII activity was consistent for all participants, the magnitude of FVIII activity was variable between participants, and predictors of response to gene therapy are still unknown.

Mean treated bleeding rate declined sharply after infusion for both cohorts over 7 years in this study, suggesting that the efficacy of valoctocogene roxaparvovec is durable in most participants (bleeding rate data included participants who resumed prophylaxis) [14]. Compared with baseline, the magnitude of decline in mean treated ABR was higher in the 6 × 10^13^ vg/kg cohort than in the 4 × 10^13^ vg/kg cohort across the 7‐year evaluation period [14]; mean FVIII infusion rates declined similarly in both cohorts [14]. In the 6 × 10^13^ vg/kg cohort, 3 of 7 participants experienced 0 treated bleeds over 7 years after receiving this higher dose of valoctocogene roxaparvovec [14], which was further evaluated in the phase 3 GENEr8‐1 trial and subsequently approved for the treatment of severe HA [7, 8, 13].

Both the mean treated ABR and the mean FVIII infusion rates were influenced by the minority of participants who resumed prophylaxis, whose bleed frequency and FVIII use were higher than other participants. Specifically in year 7, there were 20 bleeds total in the 4 × 10^13^ vg/kg cohort across 3 participants, 14 of which occurred in 1 participant who resumed routine FVIII prophylaxis during week 335. Mean ABR and FVIII infusion rates were also improved in GENEr8‐1, although to a slightly lower extent by the end of year 4 when treated ABR had declined 82.6% and annualized FVIII infusion rate had declined 95.5%. The ABRs in that study are likely a more reliable indicator of long‐term haemostatic efficacy based on the larger sample size. Because a central goal of HA treatment is to prevent bleeding, the long‐term robust improvement in bleeding rates in both cohorts may be relevant to discussions between patients and providers regarding treatment options, including gene therapy [1].

The resumption of prophylaxis with FVIII or emicizumab was tracked through the trial, and the relationship with FVIII activity and bleeding events was evaluated. Overall, 4 participants (2 in each cohort) resumed prophylaxis between years 4 and 7. Some participants declined or delayed return to prophylaxis despite FVIII activity or bleeding rates that would typically warrant prophylaxis. This observation may reflect a personal preference for, or adaptation to, new lifestyles that are free of routine infusions in a subset of participants. As described previously, a participant in the 4 × 10^13^ vg/kg cohort who resumed prophylaxis during year 5 found adherence to a regular regime difficult and switched to on‐demand FVIII use, including targeted prophylaxis as needed, after 1 month [11]. As in previous years, return to prophylaxis was discussed with all participants who had low FVIII activity as part of a shared decision‐making process.

The small sample size and inclusion of the participants who resumed prophylaxis precludes conclusive evaluation of HRQOL outcomes, which was not a primary objective of this study. However, the results from this trial suggest that improving haemostasis with a single infusion may improve HRQOL [1, 2], as mean Haemo‐QOL‐A Total Scores increased in the 6 × 10^13^ vg/kg cohort. HRQOL outcomes in the larger GENEr8‐1 population have been published previously and generally support improved HRQOL after valoctocogene roxaparvovec therapy [13, 15, 21].

The durability of valoctocogene roxaparvovec efficacy without the need for routine treatments, which lasted the entire 7‐year study for most participants, is an important factor for shared decision‐making between physicians and individuals with HA [22, 23]. Lifestyle considerations, including the desired amount of physical activity, frequency of travel, or inability to self‐treat due to disability, could be factors in whether people with HA choose to undergo gene therapy or resume prophylaxis after receiving gene therapy. Shared decision‐making is especially important when deciding to undergo an irreversible therapy. Safety‐related factors to consider are the risks of elevated liver enzymes and consequent need for immunosuppressant use after infusion. Additionally, current technology precludes receiving another AAV vector‐based gene therapy, although in the future, alternative modalities may be employed that allow redosing. Resources are available to enhance awareness and facilitate the shared decision‐making process.

As valoctocogene roxaparvovec and other haemophilia AAV gene therapies become available, long‐term follow‐up and monitoring of people with haemophilia who have received gene therapy will be essential to providing a holistic picture of the effectiveness, safety, and benefits to HRQOL. Full and minimum dataset standards have been recommended by the International Society on Thrombosis and Haemostasis Scientific and Standardization Committee, in collaboration with the World Federation of Haemophilia (WFH), to facilitate this process [24, 25]. As clinical experience grows, new benefits, concerns, or questions related to gene therapy for haemophilia may be identified. The WFH is tracking outcomes through a global Gene Therapy Registry [26].

## 5 Conclusions

Valoctocogene roxaparvovec increased FVIII activity and provided improved haemostasis compared with exogenous FVIII during baseline for most participants over 7 years. Efficacy was higher with the 6 × 10^13^ vg/kg dose than with the 4 × 10^13^ vg/kg dose. The frequency of treatment‐related AEs declined sharply after year 1. Most events were manageable, and no long‐term safety concerns were identified.

## Funding

Funding for this trial was provided by BioMarin Pharmaceutical Inc.

## Ethics Statement

All procedures were performed in accordance with the Declaration of Helsinki and Good Clinical Practice Guidelines. All participants provided written informed consent.

## Conflicts of Interest

Priyanka Raheja has received grant/travel support from CSL Behring, Roche, and Sobi and advisory honoraria from BioMarin Pharmaceutical Inc., CSL Behring, LFB Biopharmaceuticals, Pfizer, Sobi, and Takeda. Savita Rangarajan received grants from Roche and Sangamo, travel support from Reliance Life Sciences and Shire/Takeda, and consulting payments from Pfizer, Reliance Life Sciences, Sanofi, and Shire/Takeda. Will Lester received grants from BioMarin Pharmaceutical Inc., personal fees from Bayer, Chugai, Grifols, LFB Biopharmaceuticals, Novo Nordisk, Pfizer, Sobi, and Takeda, and travel support from CSL Behring and Takeda. Bella Madan has received speaker fees from BioMarin Pharmaceutical Inc. Glenn F. Pierce received consulting payments from Frontera Therapeutics, Inovio, Novo Nordisk, Roche, Sanofi, SOBI, and Third Rock Ventures and is a board member of Be Bio, the Medical and Scientific Advisory Council of the US National Bleeding Disorders Foundation, Metagenomi, Typewriter, Voyager Therapeutics, and the World Federation of Haemophilia. Emily Symington received grants from BioMarin Pharmaceutical Inc. and travel support from CSL Behring and Novo Nordisk. Konstantia‐Maria Chavele, Dane Osmond, and Mingjin Li are employees and shareholders of BioMarin Pharmaceutical Inc. Carolyn Millar has received research support from Baxter/Takeda, CSL Behring, and Grifols and honoraria or consultation fees from CSL Behring, LFB Biopharmaceuticals, Octapharma, and Takeda. She has participated in advisory boards for CSL Behring and Takeda.

## Supporting information




**Supplementary File1**: hae70284‐sup‐0001‐SuppMat.docx

## Data Availability

The de‐identified individual participant data that underlie the results reported in this article (including text, tables, figures, and appendices) will be made available together with the research protocol and data dictionaries, for non‐commercial, academic purposes. Additional supporting documents may be available upon request. Investigators will be able to request access to these data and supporting documents via a data sharing portal beginning 6 months and ending 2 years after publication. Data associated with any ongoing development program will be made available within six (6) months after approval of relevant product. Requests must include a research proposal clarifying how the data will be used, including proposed analysis methodology. Research proposals will be evaluated relative to publicly available criteria available at https://www.biomarin.com/publication‐data‐request/ to determine if access will be given, contingent upon execution of a data access agreement with BioMarin Pharmaceutical Inc.
