# Supplementary material for: Final Analysis of the Phase 1/2 Trial of Valoctocogene Roxaparvovec for Severe Haemophilia A
Source: Haemophilia. 2026 Apr 12;32(4):974–81. doi: 10.1111/hae.70284 (PMC13378670; doi:10.1111/hae.70284)
Supplement: Supplementary file 1 — Supplementary File1: hae70284‐sup‐0001‐SuppMat.docx [file HAE-32-974-s001.docx]

# Supplemental figures

**Figure S1.** Change from baseline Haemo-QOL-A domain scores in the 4x10^13^ vg/kg cohort. **A)** Physical Functioning, **B)** Role Functioning, **C)** Consequences of Bleeding, **D)** Worry, **E)** Emotional Impact, and **F)** Treatment Concern.

**A)**

**
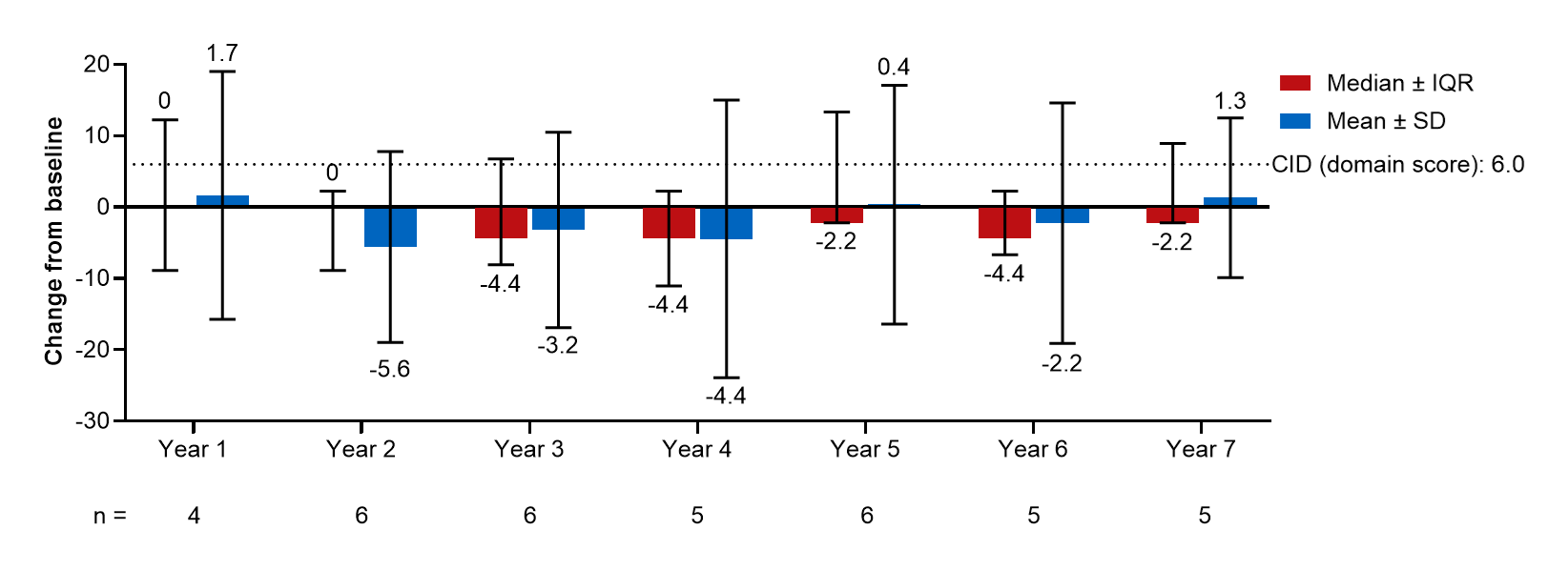
**

**B)**

**
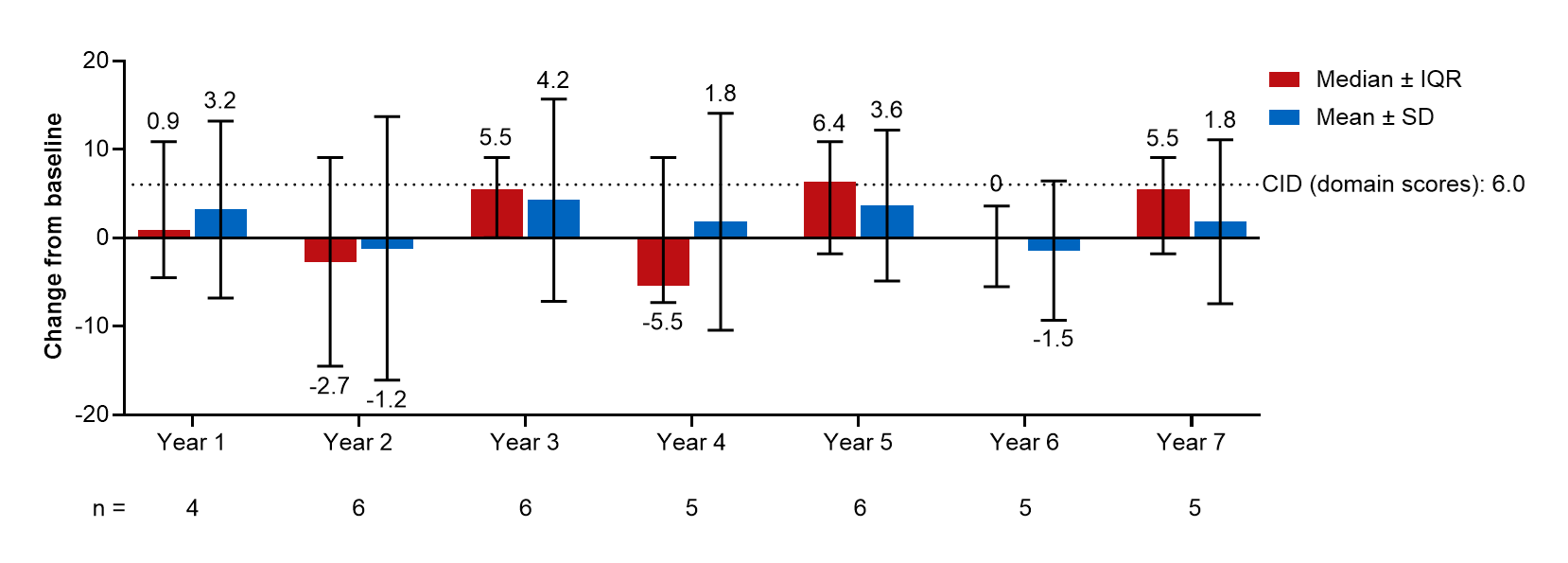
**

**C)**

**
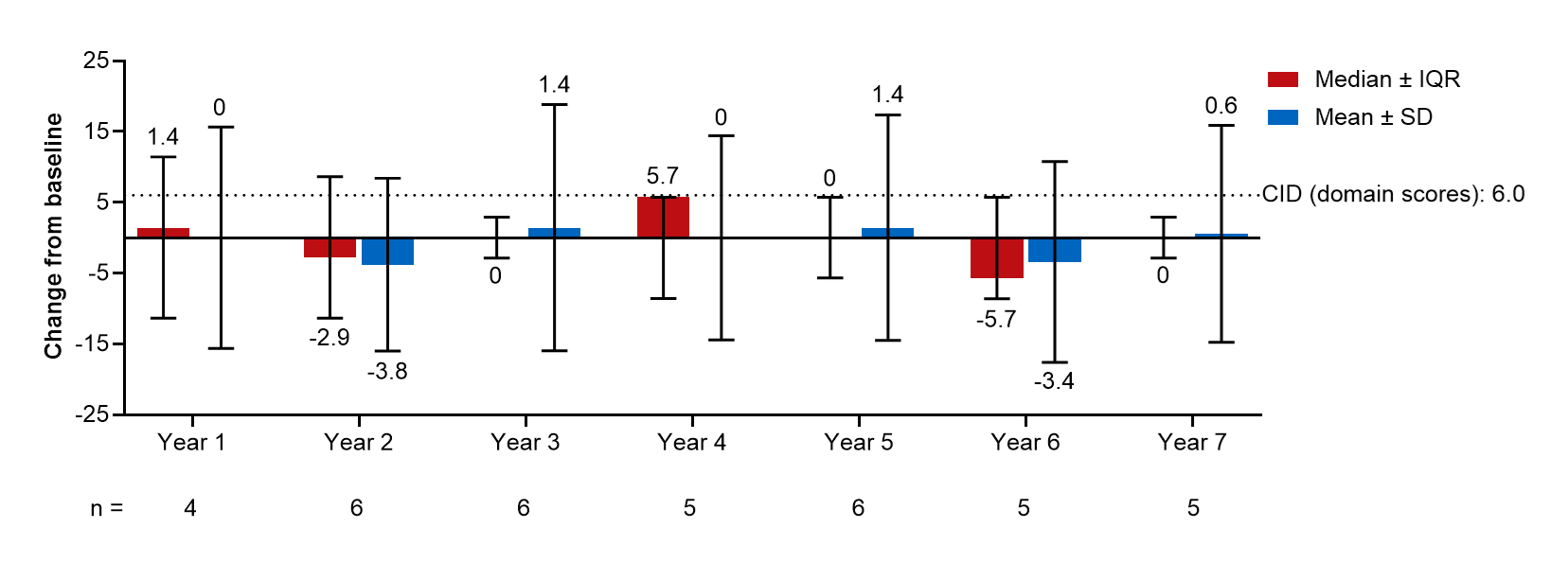
**

**D)**

**
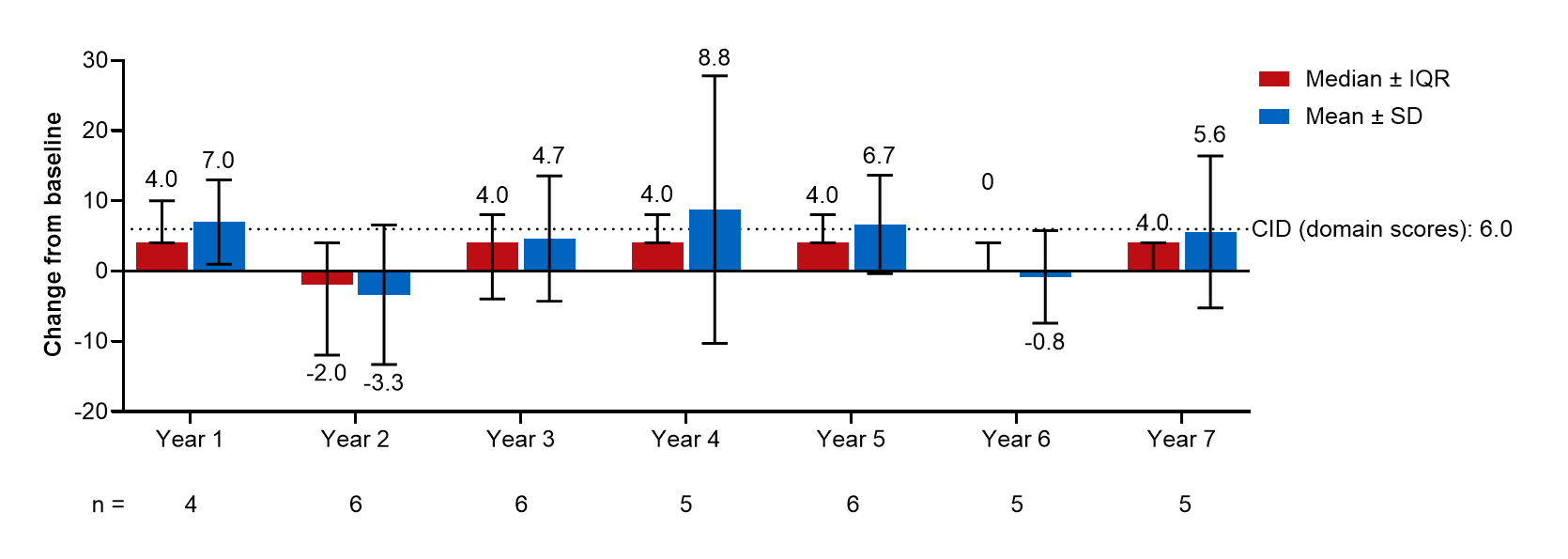
**

**E)**

**
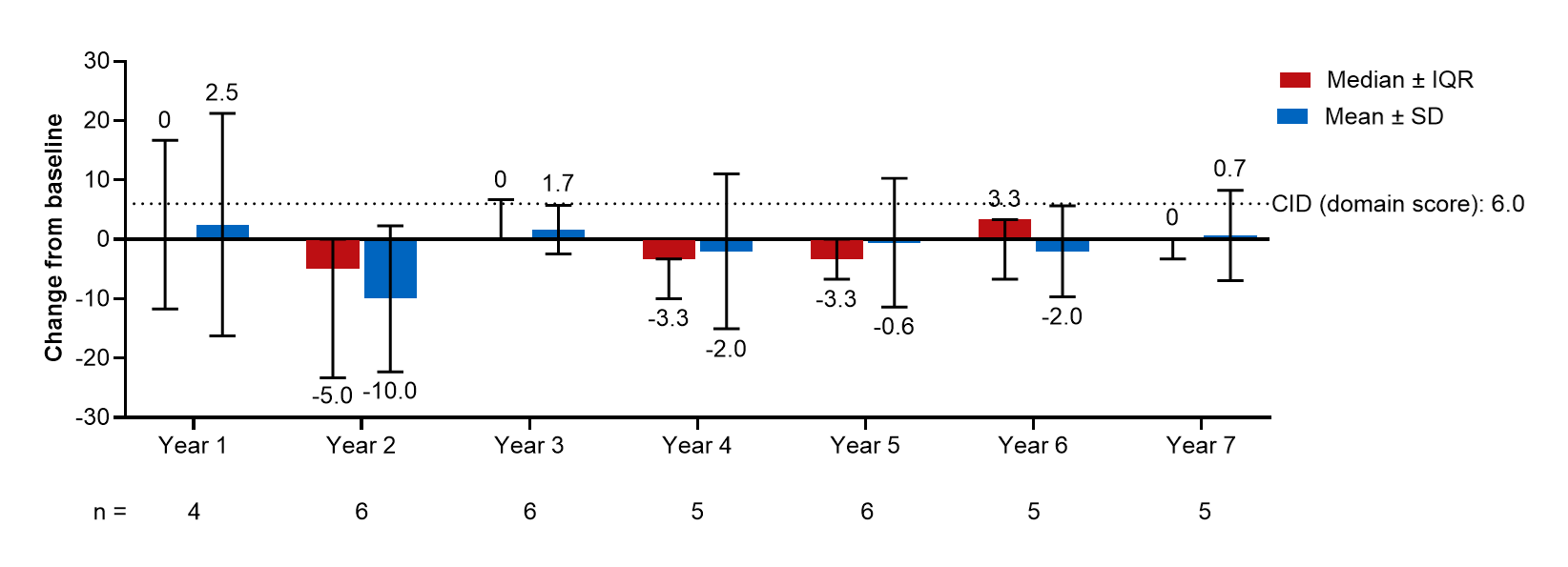
**

**F)**

**
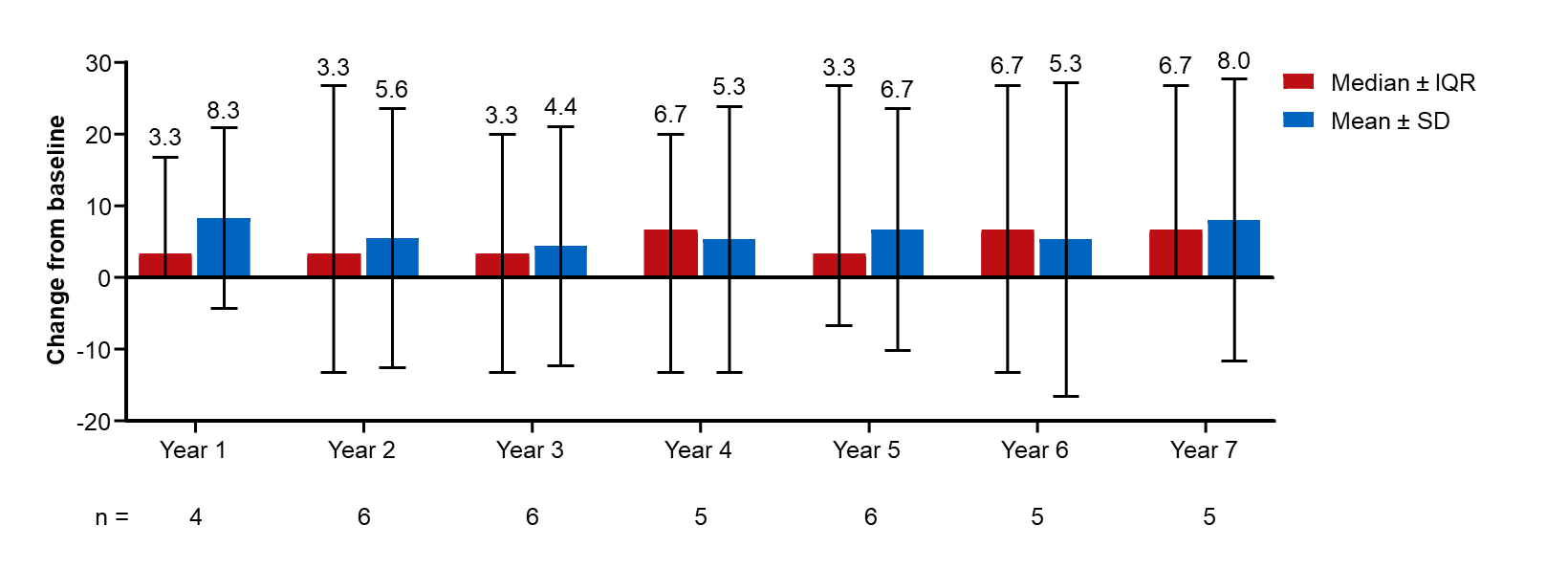
**

Data included participants who resumed prophylaxis.

CID, clinically important difference; Haemo-QOL-A, Haemophilia-Specific Quality of Life Questionnaire for Adults; IQR, interquartile range; SD, standard deviation.

**Figure S2.** Change from baseline Haemo-QOL-A domain scores in the 6x10^13^ vg/kg cohort. **A)** Physical Functioning, **B)** Role Functioning, **C)** Consequences of Bleeding, **D)** Worry, **E)** Emotional Impact, and **F)** Treatment Concern.

**A)**

**
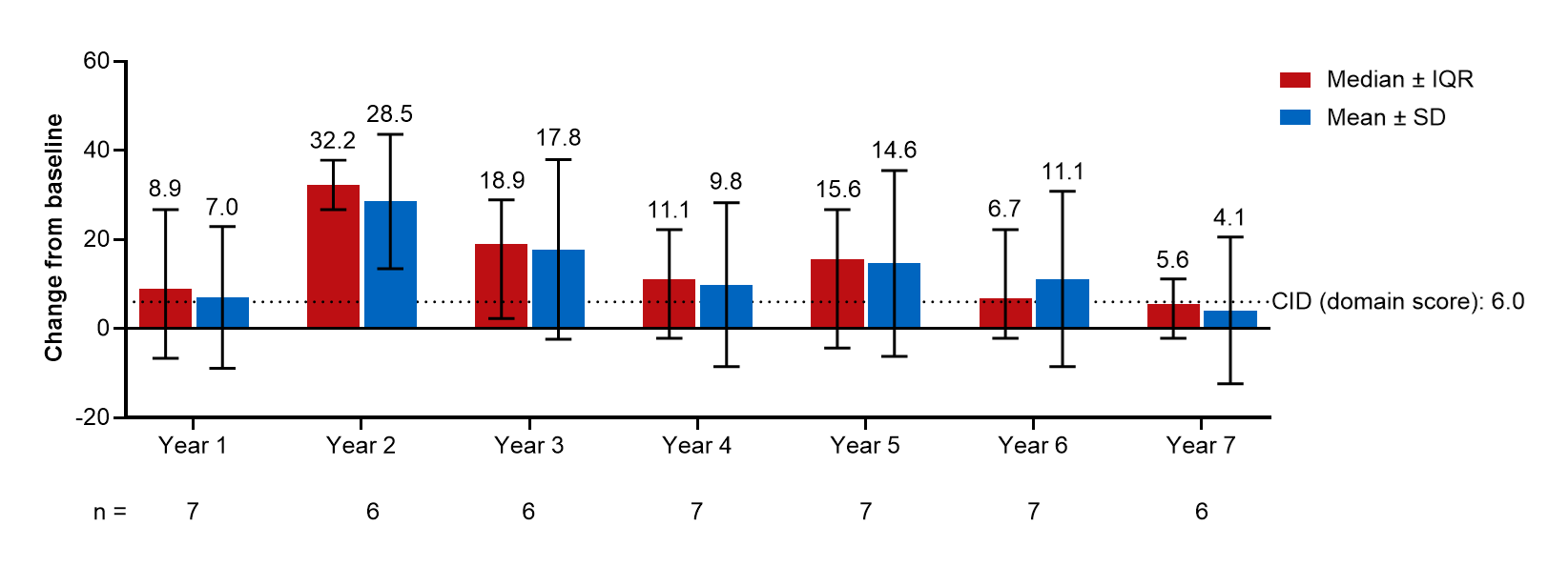
**

**B)**

**
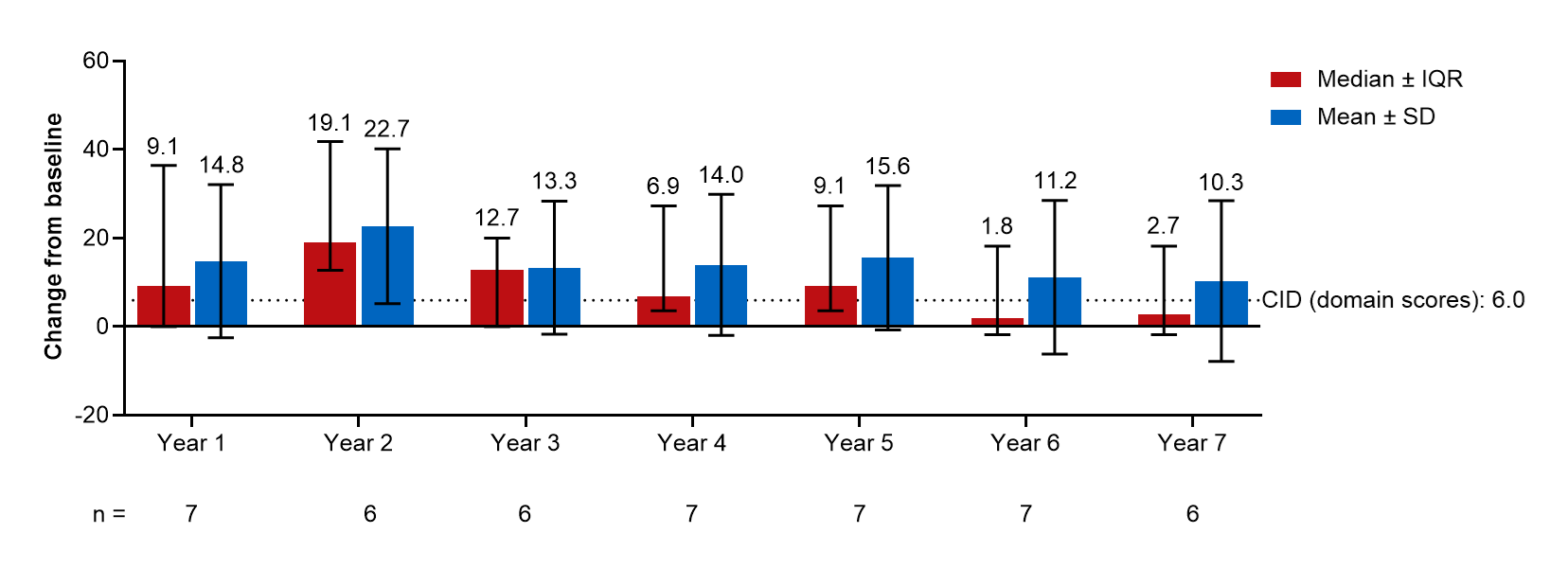
**

**C)**

**
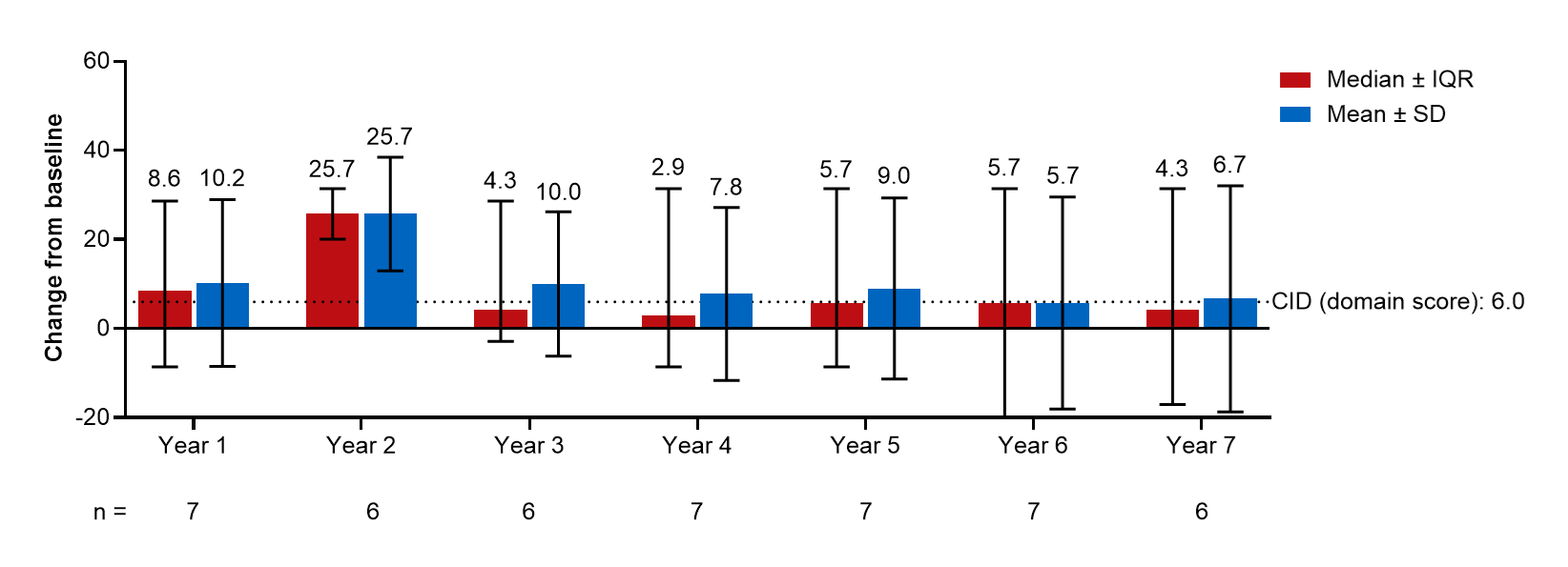
**

**D)**

**
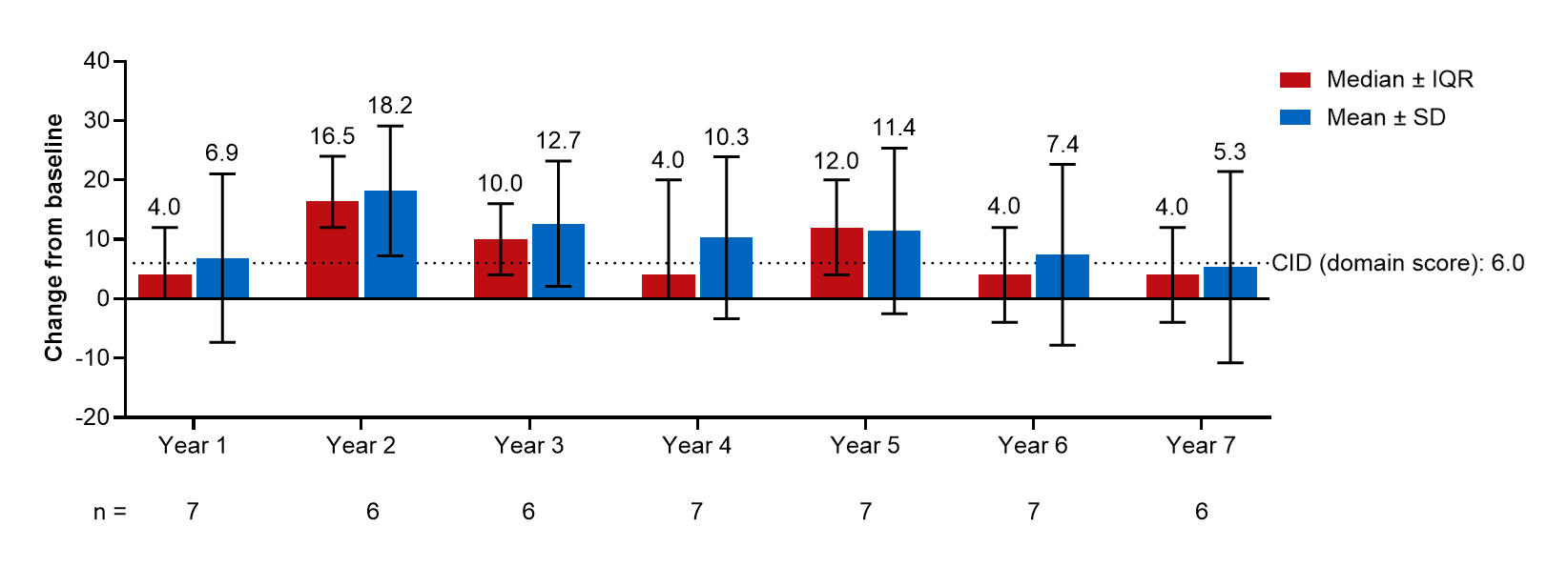
**

**E)**

**
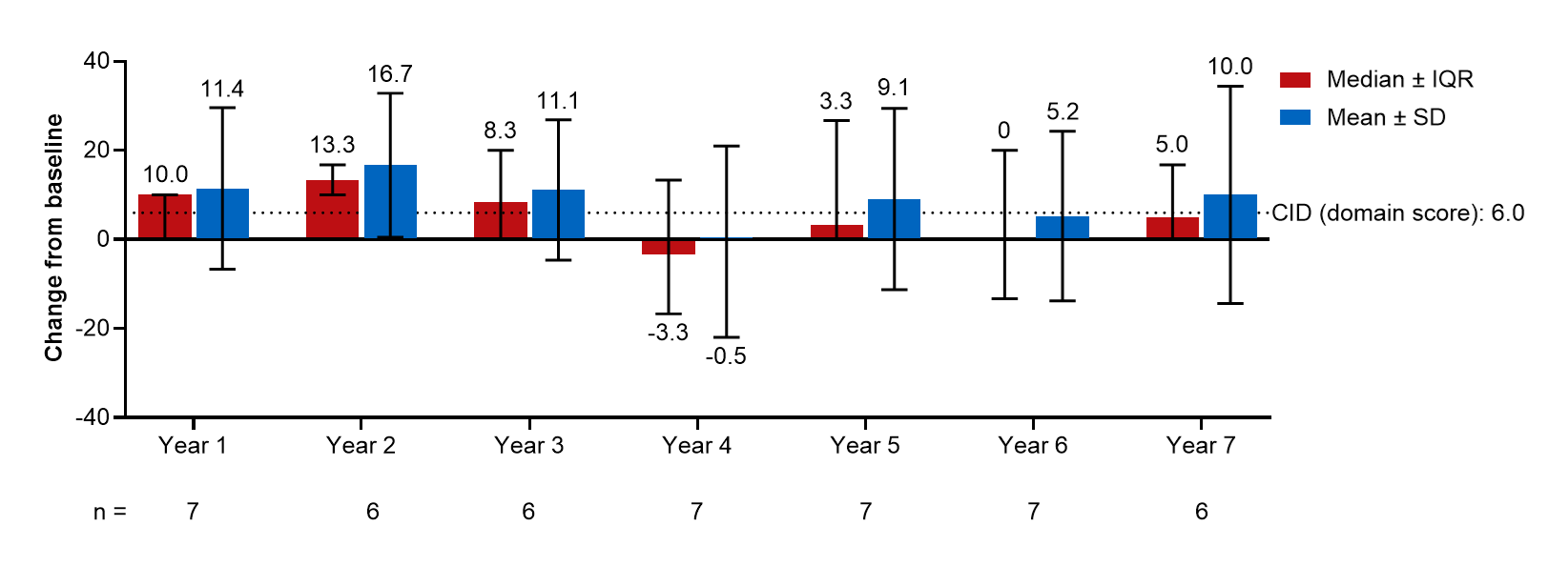
**

**F)**

**
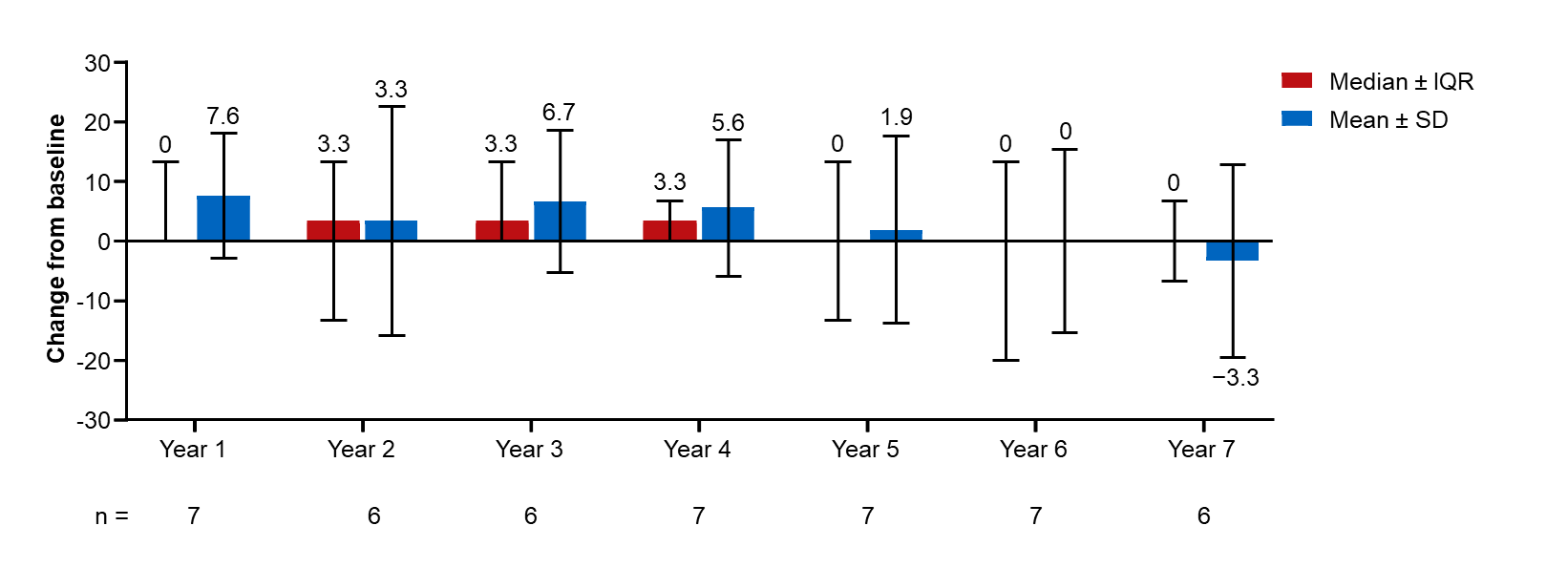
**

Data included participants who resumed prophylaxis.

CID, clinically important difference; Haemo-QOL-A, Haemophilia-Specific Quality of Life Questionnaire for Adults; IQR, interquartile range; SD, standard deviation.

# Supplemental tables

**Table S1.** Individual outcomes for participants over 7 years

| **Participant** | | **Baseline** | | **Year 1** | | **Year 2** | | **Year 3** | | **Year 4** | | **Year 5** | | **Year 6** | | **Year 7** | | **Week 5 through end of study period** | |
| --- | --- | --- | --- | --- | --- | --- | --- | --- | --- | --- | --- | --- | --- | --- | --- | --- | --- | --- | --- |
|  |  | **ABR (#/year)** | **AFR (#/year)** | **FVIII (IU/dL)** | **# bleeds** | **FVIII (IU/dL)** | **# bleeds** | **FVIII (IU/dL)** | **# bleeds** | **FVIII (IU/dL)** | **# bleeds** | **FVIII (IU/dL)** | **# bleeds** | **FVIII (IU/dL)** | **# bleeds** | **FVIII (IU/dL)** | **# bleeds** | **ABR (#/year)** | **AFR (#/year)** |
| **6x10^13^ vg/kg** | **3** | 9.0 | 119.8 | 62.1 | 0 | 26.2 | 0 | 10.8* | 0 | BLOQ | 0 | 2.5 | 0 | 5.6 | 3 | 4.0 | 0 | 0.4 | 5.7 |
|  | **4** | 25.0 | 27.4 | 49.3 | 1 | 24.6 | 0 | 19.9* | 0 | 14.5 | 1 | 8.2 | 0 | 4.0 | 1 | 4.8 | 1 | 0.6 | 1.6 |
|  | **5** | 1.0 | 151.7 | 94.6 | 0 | 38.2 | 0 | 37.6* | 0 | 18.0 | 0 | 14.1 | 0 | 13.4 | 0 | 10.3 | 0 | 0 | 0.1 |
|  | **6** | 24.0 | 158.5 | 10.6 | 7 | 3.9 | 1 | 4.1* | 4 | 3.1* | 8 | 1.8 | 4 | 1.9 | 1 | RTP^a^ | 3 | 3.9 | 20.0 |
|  | **7** | 40.0 | 157.1 | 88.4 | 0 | 86.0 | 0 | 100.1 | 0 | 62.2 | 0 | 35.0 | 0 | 28.0 | 0 | 47.8 | 0 | 0 | 0 |
|  | **8** | 24.0 | 121.4 | 51.9 | 0 | 24.1 | 0 | 16.5* | 0 | 11.1 | 0 | 5.0 | 0 | 5.1 | 0 | RTP^b^ | 2 | 0.3 | 15.8 |
|  | **9** | 0 | 104.9 | 54.6 | 0 | 51.7 | 0 | 36.2 | 0 | 29.5 | 0 | 19.4 | 0 | 10.8 | 0 | 14.2 | 0 | 0 | 1.5 |
| **4x10^13^ vg/kg** | **10** | 1.0 | 182.5 | 17.7 | 0 | 13.1 | 0 | 10.7 | 1 | 6.2 | 2 | 5.0 | 0 | 2.9 | 0 | 1.8 | 1 | 0.6 | 2.2 |
|  | **11** | 41.0 | 184.3 | 40.1 | 0 | 20.1 | 0 | 22.4 | 0 | 13.7 | 0 | 9.6 | 1 | 9.4 | 16 | RTP^c^ | 14 | 4.5 | 14.0 |
|  | **12** | 0 | 156.2 | 23.7 | 0 | 10.0 | 0 | 3.9 | 0 | BLOQ | 2 | 6.4 | 1 | 6.0 | 0 | 2.0 | 0 | 0.4 | 12.7 |
|  | **13** | 4.0 | 53.8 | 14.3 | 0 | 7.9 | 0 | 3.4 | 0 | 3.0 | 0 | 2.1 | 0 | LTF | LTF | - | - | 0 | 3.7 |
|  | **14** | 15.0 | 124.8 | 25.5 | 0 | 22.4 | 1 | 20.5 | 0 | 10.5 | 0 | 10.1 | 1 | 8.4 | 0 | 8.8 | 0 | 0.3 | 3.4 |
|  | **15** | 12.0 | 155.5 | BLOQ | 5 | BLOQ* | 6 | BLOQ | 2 | 2.5 | 6 | RTP^d^ | 1 | NA | 0 | NA | 5 | 3.7 | 25.6 |

Return to prophylaxis study week: ^a^Week 361 with emicizumab; ^b^Week 338 with octocog alfa; ^c^Week 335 with turoctocog alfa pegol; ^d^Week 240 with turoctocog alfa pegol, for 1 month.

*The measurement displayed is within 4 weeks of the study week associated with that year.

FVIII measurements are using the chromogenic substrate assay and from the end of the year. Bleeds are treated bleeds and are counted cumulatively across the entire year (ie, from week 53 to week 104). ABR and AFR from week 5 (when prophylaxis was scheduled to end) through the end of the study period or when participants were lost to follow-up.

ABR, annualized bleeding rate; AFR, annualized FVIII infusion rate; BLOQ, below limit of quantification; FVIII, factor VIII; LTF, lost to follow-up; NA, not available; RTP, return to prophylaxis
